# Reflection Reduction on DDR3 High-Speed Bus by Improved PSO

**DOI:** 10.1155/2014/257972

**Published:** 2014-03-18

**Authors:** Huiyong Li, Hongxu Jiang, Bo Li, Miyi Duan

**Affiliations:** Beijing Key Laboratory of Digital Media, School of Computer Science and Engineering, Beihang University, Beijing 100191, China

## Abstract

The signal integrity of the circuit, as one of the important design issues in high-speed digital system, is usually seriously affected by the signal reflection due to impedance mismatch in the DDR3 bus. In this paper, a novel optimization method is proposed to optimize impedance mismatch and reduce the signal refection. Specifically, by applying the via parasitic, an equivalent model of DDR3 high-speed signal transmission, which bases on the match between the on-die-termination (ODT) value of DDR3 and the characteristic impedance of the transmission line, is established. Additionally, an improved particle swarm optimization algorithm with adaptive perturbation is presented to solve the impedance mismatch problem (IPSO-IMp) based on the above model. The algorithm dynamically judges particles' state and introduces perturbation strategy for local aggregation, from which the local optimum is avoided and the ability of optimization-searching is activated. IPSO-IMp achieves higher accuracy than the standard algorithm, and the speed increases nearly 33% as well. Finally, the simulation results verify that the solution obviously decreases the signal reflection, with the signal transmission quality increasing by 1.3 dB compared with the existing method.

## 1. Introduction

As the most popular memory, DDR3 illustrates faster speed, higher data rate, and lower operating voltages than DDR2, with the data rate up to 1.6 Gbps or even higher at the operating voltage of 1.5 V. However, DDR3 memory requires more in its interconnect interface design while bringing higher data transmission rate. During the high-speed signal transmission, sudden changes of transient impedance will lead to discontinuity of signal line impedance, which results in signal reflections and thus substantial overshoot, undershoot, and ringing. Therefore, researching the signal reflection of DDR3 bus has become a key component in the design of high-speed digital system.

There has been considerable research on optimization of DDR3 bus signal quality. Jagdale et al. [1] researched on the signal reflection in high-speed PCB design and discussed several main factors leading to discontinuous signal. The conducted experiments concluded that the ODT and characteristic impedance of transmission line play an important role in reducing signal reflection. Considering the influence of power consumption, timing, and voltage amplitude, Mintarno and Ji [2] proved that in high-speed memory bus design, ODT optimization can substantially reduce power consumption while drastically improve the signal integrity. For the multiple DDR3 modules extension in computer motherboard, Lin et al. [3, 4] applied particle swarm optimization (PSO) algorithm to motherboard routing and selection of ODT value, taking into account both impedance discontinuity caused by multiple ports and crosstalk caused by adjacent transmission lines. The experiments show that transmission quality of signal is enhanced while signal reflection is reduced.

The research above shows that in DDR3 bus design, the discontinuity of characteristic impedance is the primary factor causing signal reflection, which indicates that an appropriate ODT value could effectively decrease impedance discontinuity of signal transmission line. Jagdale et al. [1] and Mintarno and Ji [2] verified the effort of ODT value, which is of important significance in this field, yet the specific optimization strategies are not explicitly given. Thus, the key point in high-speed bus design of DDR3 is how to accurately evaluate the impedance matching between ODT value and routing. Through theoretical derivation, Lin et al. [3, 4] proposed an approach to optimize ODT and routing parameters using PSO, which obtains ideal results. Nevertheless, such researches usually attach importance to the effects of transmission line itself on the signal but neglect discontinuity problems caused by the via. Via is a typical discontinuity for high-speed signal transmission in printed circuit boards [5, 6]. The via basically does not affect signal transmission in low frequency. Nevertheless, as the frequency goes up to GHz, the impact of the via on signal integrity must be considered [7].

Multiple factors being considered are bound to bring greater difficulties in the circuit optimization. Particle swarm optimization (PSO) [8], as a random search algorithm based on group cooperation, could be used to solve multidimensional complex optimization problems in various fields [9]. Each particle in the swarm represents a possible solution to the optimization problem. During the calculation of *K* iterations, particle constantly adjusts its position according to local optimal solution obtained from its motion and global optimal solution obtained from group interaction and gradually closes to the optimal solution [10]. The PSO algorithm introduced in [4] provided a feasible scheme to solve the optimization problem of multiple parameters. However, as an evolutionary optimization algorithm, the standard PSO has two operations, exploration and exploitation, which makes it easy to involve into local optimum with the iterations increasing. And consequently, the convergence speed is reduced.

Li et al. [11] proposed an enhanced PSO algorithm specifically for electromagnetic field. To increase the convergence speed, the PSO algorithm is improved in many aspects, including the perturbation strategy for global optimal value to avoid the local optimum. Yet, the solution reduces the initial efficiency to some extent. Melin et al. [12] proposed an improvement to the convergence and diversity of the swarm in PSO using fuzzy logic, which improves the performance of PSO. Maldonado et al. [13] described the design of a type-2 average fuzzy system on FPGA and its optimization using particle swarm optimization for the speed regulation of a DC motor, which implements the PSO optimization of interval tye-2 fuzzy controllers for FPGA applications. By analyzing the movement behavior of the particles, Ying-Qiu et al. [14] utilized a method, which dynamically adjusts the boundary of search space, to trace the particle position in order to prevent particles from gathering locally. This strategy relieves the premature convergence while maintaining initial efficiency, improving the accuracy of algorithm. But the algorithm adopts a random processing mechanism to handle the stagnating particles in which possibility brings some uncertain factors to optimization process and it is easy to introduce invalid operations.

In this paper, how to improve the efficiency while maintaining initial high performance is the key point. The major contributions of this paper are as follows: (1) an improved optimization strategy, which combines the characteristics of embedded hardware, is proposed in this paper for design of DDR3 high-speed bus. The strategy considers the impact of via on high-speed signal; (2) we propose an improved particle swarm optimization algorithm with adaptive perturbation, which optimizes routing length of DDR3 signal, characteristic impedance, via parasitic impedance, and the impact of ODT on high-frequency signal quality; (3) the experiments demonstrate that the strategy proposed could improve effectively impedance continuity of transmission line and reduce reflection effects of high-speed signals.

The rest of this paper is organized as follows. In Section 2, the transmission line and via model are described. In Section 3, firstly, by applying via parasitic, an equivalent model of DDR3 high-speed signal transmission is presented. Then, an improved particle swarm optimization algorithm with adaptive perturbation is presented to solve the impedance mismatch problem (IPSO-IMp) based on the above model. The experimentation and simulation are shown in Section 4, and the conclusions are finally summarized in Section 5.

## 2. Transmission Line and Via Model

### 2.1. Transmission Line Model

**Figure 1:**
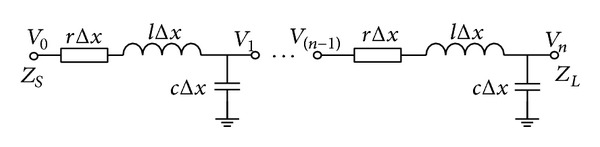
Transmission line equivalent model.

In high frequency, interconnection lines exhibit characteristics of transmission lines. Thus, distributed model of lossy transmission line cascaded by *n* RLCs can be used to approximate interconnection lines, as shown in Figure 1, where *r*, *l*, and *c*, respectively, represent resistance, inductance, and capacitance of transmission line per unit length and *Z*_*S*_ and *Z*_*L*_ denote the driver impedance and load impedance, respectively.

The driver and the load do not have signal reflection when *Z*_*S*_ = *Z*_*L*_ = *Z*_0_, which is difficult to achieve in practical applications. Actually, there exists reflected signal in interconnection line, which leads to overshoot and undershoot on output voltage. The overshoot decreases the stability of the circuit, while undershoot may lead to slight pulse interference, aggravating energy dynamic distribution and even false triggering, which may result in serious logical error and timing error.

### 2.2. Via Model

Via is the conductor connecting the lines of different signal layers in multilayer PCB. Studies [5, 15, 16] have shown that both via diameter and pad size affect impedance continuity.

**Figure 2:**
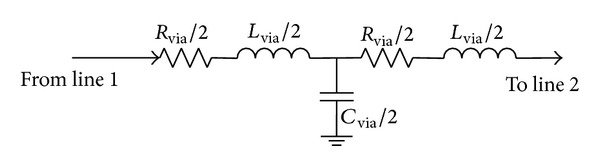
Via equivalent model.

Figure 2 shows via equivalent model, where *R*_via_, *L*_via_, and *C*_via_, respectively, represent the parasitic resistance, parasitic inductance, and parasitic capacitance. Their values mainly depend on via radius *r*_via_ and via length *h*_via_, with the specific calculation formula given in [17].

**Figure 3:**
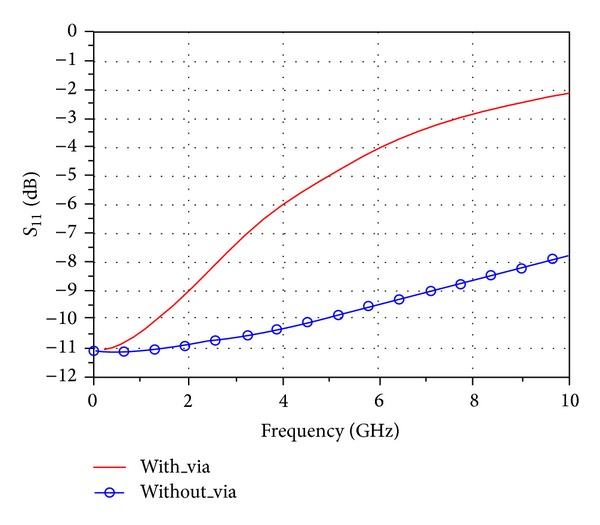
The impact of the via on signal reflection.

Since parasitic capacitance, parasitic inductance, and parasitic resistance exist in via, via leads to impedance discontinuities on high-frequency signal transmission line, which results in signal reflection.  Figure 3 illustrates via effects on signal reflection.

It is easily seen that via effects on signal reflection are even obvious when the frequency exceeds 1 GHz. The line between DSP processor and DDR3 pins inevitably has at least two vias, and the data rate can reach 1.6 Gb/s or even higher; therefore, the effect of via's parasitic resistance, parasitic inductance, and parasitic capacitance on high frequency signal cannot be ignored. And the discontinuity occurred leads to issue of signal reflection, which should be considered in design of DDR3 high-speed bus interconnection [17].

## 3. Proposed Methodology

### 3.1. High-Speed Bus Structure and Model

**Figure 4:**
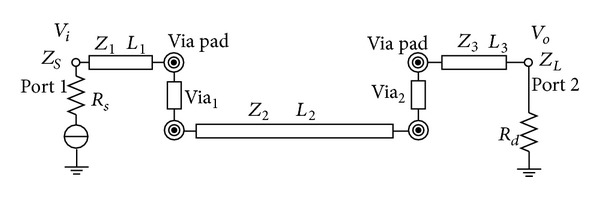
High-speed transmission line structure.

In this section, transmission coefficient and reflection coefficient for DDR3 bus transmission line are obtained through parameter *S*. For general design, we suppose that transmission line has two vias and Figure 4 shows high-speed interconnect structure between DSP processor and DDR3. For simplicity, the parameters are denoted as follows:

- the two vias connecting the three transmission lines are presented by Via_1_ and Via_2_;
- the length of the three transmission lines is, respectively, denoted by *L*_1_, *L*_2_, and *L*_3_;
- the characteristic impedance of the three transmission lines is, respectively, denoted by *Z*_1_, *Z*_2_, and *Z*_3_;
- the internal impedance of the source and load is presented by *Z*_*S*_ and *Z*_*L*_;
- the power of source end and load end is, respectively, denoted by *V*_*i*_ and *V*_*o*_.

**Figure 5:**
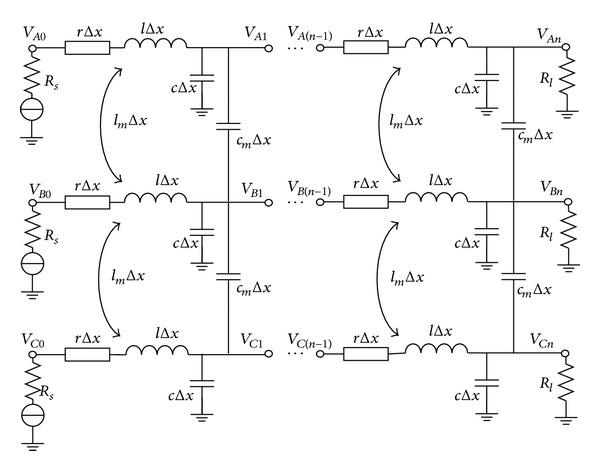
Driver line and victim line model.

According to the electromagnetic theory, each transmission line is subject to interference from surrounding transmission lines, especially the adjacent lines. Therefore, we take three adjacent transmission lines as study object in this paper in order to approximate the actual circuit, provided that (1) the length of a transmission line is *L* and (2) resistance, inductance, capacitance, mutual inductance, and mutual capacitance for per unit length is *r*, *l*, *c*, *l*_*m*_, and *c*_*m*_, respectively. According to transmission lines equivalent model given in Figure 2, the driver and victim transmission model could approximate to the model shown in Figure 5, where *A* and *C* represent driver lines and *B* represents victim line, respectively.

The first RLC unit of victim line is analyzed first. According to the basic circuit theorem, we obtain the following:

(1) VB0(s)=(r+sl)ΔxIB0(s)+slmΔxIA0(s)+slmΔxIC0(s)+VB1(s),

(2) IB0(s)=scΔxVB1(s)+scmΔx(VB1(s)−VA1(s))+scmΔx(VB1(s)−VC1(s))+IB1(s),

(3) $$\begin{array}{c} V_{A0}\left(s\right)=\left(r+sl\right)\Delta xI_{A0}\left(s\right)+sl_{m}\Delta xI_{B0}\left(s\right)+V_{A1}\left(s\right), \end{array}$$

(4) $$\begin{array}{c} V_{C0}\left(s\right)=\left(r+sl\right)\Delta xI_{C0}\left(s\right)+sl_{m}\Delta xI_{B0}\left(s\right)+V_{C1}\left(s\right). \end{array}$$

When *V*_*A*1_ = *V*_*C*1_ = *KV*_*B*1_, *V*_*A*0_ = *V*_*C*0_ = *KV*_*B*0_, by integrating (1), (2), (3) and (4) the *V*_*B*0_ and *I*_*B*0_ could be defined as

(5) IB0(s)=[c+2cm(1−K)]sΔxVB1(s)+IB1(s),VB0(s)=(1+(r+sl)2−2(slm)2r+s(l−2Klm) ×Δx[c+2cm(1−K)]sΔx)VB1(s)+(r+sl)2−2(slm)2r+s(l−2Klm)ΔxIB1(s).

When *a* = (((*r*+*sl*)^2^ − 2(*sl*_*m*_)^2^)/(*r* + *s*(*l* − 2*Kl*_*m*_)))Δ*x*, *b* = [*c* + 2*c*_*m*_(1 − *K*)]*s*Δ*x*, according to (5), we get *V*_*B*0_ and *I*_*B*0_ as follows:

(6) $$\begin{array}{c} I_{B0}\left(s\right)=bV_{B1}\left(s\right)+I_{B1}\left(s\right),\\ V_{B0}\left(s\right)=\left(1+ab\right)V_{B1}\left(s\right)+aI_{B1}\left(s\right). \end{array}$$

The voltage and current of victim lines could be expressed as

(7) $$\begin{array}{c} \left[\begin{array}{c} V_{B0}\left(s\right)\\ I_{B0}\left(s\right) \end{array}\right]=\left[\begin{array}{cc} 1+ab & a\\ b & 1 \end{array}\right]\left[\begin{array}{c} V_{B1}\left(s\right)\\ I_{B1}\left(s\right) \end{array}\right]. \end{array}$$

Formula (7) is an ABCD matrix revealing the relationship between input and output on the first RLC unit of victim lines. A victim line with length *L* could be seen as cascade of *n* RLC units, with the relationship between input and output demonstrated as follows:

(8) $$\begin{array}{c} \left[\begin{array}{c} V_{B0}\left(s\right)\\ I_{B0}\left(s\right) \end{array}\right]={\left[\begin{array}{cc} 1+ab & a\\ b & 1 \end{array}\right]}^{n}\left[\begin{array}{c} V_{Bn}\left(s\right)\\ I_{Bn}\left(s\right) \end{array}\right]. \end{array}$$

When *n* tends to positive infinity, parameters of matrix ABCD for the whole line are obtained from (8). We have

(9) $$\begin{array}{c} \left[\begin{array}{cc} A & B\\ C & D \end{array}\right]=\left[\begin{array}{cc} \cosh\left(\gamma_{0}L\right) & Z_{0}\sinh\left(\gamma_{0}L\right)\\ \left(\frac{1}{Z_{0}}\right)\sinh\left(\gamma_{0}L\right) & \cosh\left(\gamma_{0}L\right) \end{array}\right], \end{array}$$

where *Z*_0_ and *γ*_0_ are, respectively, characteristic impedance and propagation constant of transmission lines when mutual interaction is considered. And they are expressed as follows:

(10) $$\begin{array}{c} Z_{0}=\sqrt{\frac{a}{b}}=\sqrt{\frac{{\left(r+sl\right)}^{2}-2{\left(sl_{m}\right)}^{2}}{\left[r+s\left(l-2Kl_{m}\right)\right]\left[c+2c_{m}\left(1-K\right)\right]s}},\\ \gamma_{0}=\frac{\sqrt{ab}}{\Delta x}=\sqrt{\frac{{\left(r+sl\right)}^{2}-2{\left(sl_{m}\right)}^{2}}{\left[r+s\left(l-2Kl_{m}\right)\right]}\left[c+2c_{m}\left(1-K\right)\right]s}. \end{array}$$

Given that (1) the via impact on high-speed bus is considered, (2) the impact among vias is ignored, and (3) Figure 4 is replaced by via equivalent model in Figure 2 and transmission line equivalent model in Figure 1, the relationship between input and output for the entire high-speed line could be expressed as follows:

(11) [Vi(s)Ii(s)]=[cosh⁡(γ1L1)Z1sinh⁡(γ1L1)(1Z1)sinh⁡(γ1L1)cosh⁡(γ1L1)] ×[1Rvia+sLvia01][10sCvia1] ×[cosh⁡(γ2L2)Z2sinh⁡(γ2L2)(1Z2)sinh⁡(γ2L2)cosh⁡(γ2L2)] ×[1Rvia+sLvia01][10sCvia1] ×  [cosh⁡(γ3L3)Z3sinh⁡(γ3L3)(1Z3)sinh⁡(γ3L3)cosh⁡(γ3L3)][Vo(s)Io(s)]=[A′B′C′D′][Vo(s)Io(s)].

The *S* parameter could be obtained by matrix ABCD. Hence, when writing DDR3, we can get from formula (11) the reflection coefficient and transmission coefficient for the whole transmission line:

(12) $$\begin{array}{c} S_{11}=\frac{A^{\prime }\sqrt{R_{l}/R_{s}}+B^{\prime }\left(1/\sqrt{R_{l}\cdot R_{s}}\right)-C^{\prime }\sqrt{R_{l}\cdot R_{s}}-D^{\prime }\sqrt{R_{s}/R_{l}}}{A^{\prime }\sqrt{R_{l}/R_{s}}+B^{\prime }\left(1/\sqrt{R_{l}\cdot R_{s}}\right)+C^{\prime }\sqrt{R_{l}\cdot R_{s}}+D^{\prime }\sqrt{R_{s}/R_{l}}},\\ S_{21}=\frac{2}{A^{\prime }\sqrt{R_{l}/R_{s}}+B^{\prime }\left(1/\sqrt{R_{l}\cdot R_{s}}\right)+C^{\prime }\sqrt{R_{l}\cdot R_{s}}+D^{\prime }\sqrt{R_{s}/R_{l}}}. \end{array}$$

Similarly, reflection coefficient *S*_12_ and transmission coefficient *S*_22_ for the whole transmission line could be gained when reading DDR3.

Equation (12) showed that the impedance matching optimization for DDR3 high-speed bus involves many parameters. If the PSO algorithm is used, we need to establish constraints on these parameters, namely, constructing fitness function.

### 3.2. Fitness Function Construction

Due to the lossy transmission, signal of sending end cannot be sent to the receiving end completely. Moreover, due to the coexistence of transmission signal and reflection signal, improving quality of signal received needs to reduce the reflection signal and enhance the transmission signal. And thus the fitness function for DDR3 bus should be defined according to both the transmission signal and the reflection signal. Provided that the transmission line is lossless when writing to DDR3, we can obtain the following according to conservation of energy:

(13) $$\begin{array}{c} {\left|S_{11}\right|}^{2}+{\left|S_{21}\right|}^{2}=1. \end{array}$$

Equation (13) indicates that signal transmitted to the receiving end achieves maximum energy when the reflection coefficient *S*_11_ tends to 0 and *S*_21_ tends to 1. Thus, fitness function could be constructed as

(14) $$\begin{array}{c} F_{W}=\left(1-{\left|S_{21}\left(f\right)\right|}^{2}\right)+{\left|S_{11}\left(f\right)\right|}^{2}. \end{array}$$

Similarly, when reading from DDR3, the fitness function could be obtained as follows:

(15) $$\begin{array}{c} F_{R}=\left(1-{\left|S_{12}\left(f\right)\right|}^{2}\right)+{\left|S_{22}\left(f\right)\right|}^{2}. \end{array}$$

We get from (14) and (15) the fitness function for both read and write states as follows:

(16) F=(1−|S21(f)|2+|S11(f)|2)+(1−|S12(f)|2+|S22(f)|2),

where *f* is the signal sampling frequency. When *f* = 2.0 GHz and (16) gains the minimum value, we can get minimum reflection signal and implement the impedance matching at 2.0 GHz. To achieve impedance matching for bandwidth sequence from 0 to 2.0 GHz, the fitness function [4] could be acquired from (16) as follows:

(17) F=∑n=0N[(1−|S21(nf0)|2+|S11(nf0)|2)  +(1−|S12(nf0)|2+|S22(nf0)|2)],

where *N* denotes frequency point chosen from 0 to 2.0 GHz. On the premise of bandwidth impedance matching, this paper takes *N* = 80 and *f*_0_ = 25 MHz to guarantee the accuracy and operation efficiency. We can get the following:

(18) F=∑n=080[(1−|S21(nf0)|2+|S11(nf0)|2)  +(1−|S12(nf0)|2+|S22(nf0)|2)].

Equation (18) is the fitness function for DDR3 signal optimization in bandwidth 0 ~ 2.0 GHz. The function contains 12 parameters, as listed in Table 1.

It should be noted that values of *L*_1_, *L*_2_, and *L*_3_ depend on the size of PCB. In this paper, the maximum value is 2000 mil and the minimum value is 0 mil. In order to facilitate the initialization of the optimization, the range of *Z*_*L*_1__, *Z*_*L*_2__, *Z*_*L*_3__, *r*_via_, and *h*_via_ is defined in Table 1. The specifications for DDR3 SDRAM were specified by the Joint Electron Device Engineering Council (JEDEC). The values of *R*_*SW*_, *R*_*LW*_, *R*_*SR*_, and *R*_*LR*_ follow the JEDEC standard.

Equation (18) could gain a minimum value by these parameters' optimization, which enables impedance matching for DDR3 bus transmission in bandwidth 0 ~ 2.0 GHz. In this case, the reflection signal is minimum and the transmission signal is maximum; namely, the optimal solution is obtained.

The above discussion tells us that the problem is transformed into the optimization of nonlinear function with multiple parameters. Exactly speaking, 12 parameters of them need to be optimized in this problem. Due to the premature convergence of standard PSO algorithm which is easy to fall into local optimal, we present an improved particle swarm optimization algorithm for solving impedance mismatch problem (IPSO-IMp).

### 3.3. IPSO for Impedance Mismatch Problem (IPSO-IMp)

In this section, the IPSO-IMp is proposed and described. By analyzing the particle movement during PSO algorithm optimization, it can be easily seen that the main reason leading to particle local optimum is that global optimal particle is too dependent on the individual optimal solution [14]. When the individual particles gather in a relatively concentrated area, the impact on the global optimal solution will become slight. Actually, the leader should be responsible for the swarm movement except for considering the individual influence. Based on this, the algorithm proposed introduces disturbance mechanism mentioned in [11] to handle particle swarm Perturbation. Yet, disturbance is carried out through the entire evolutionary process, which not only increases the computation complexity but also affects the initial evolution speed. Therefore, this paper proposes an adaptive method to judge dynamically whether particles are in local aggregation state. On basis of local aggregation, perturbation strategy is introduced to optimize the intervention, so that the local optimum is avoided and the ability of optimization-searching is activated. Furthermore, the convergence speed and accuracy are improved. The improved method we proposed is as follows.

#### 3.3.1. Particle Representation

*Position*. In the IPSO-IMp, the position of a particle is represented by

(19) $$\begin{array}{c} X_{i}=\left[x_{i}^{1},x_{i}^{2},x_{i}^{3},\ldots ,x_{i}^{12}\right], \end{array}$$

where *X*_*i*_ is defined as 12-dimensional space that is composed of the parameters in Table 1; *x*_*i*_^*n*^  (1 ≤ *n* ≤ 12) is one of the parameters and *n* is the number of the parameter. In order to ensure the local searching behaviors and the population diversity, the initial values are randomly generated based on the range in Table 1. In addition, because four parameters of 9 ≤ *n* ≤ 12 are discrete, each particle *i* is defined as

(20) $$\begin{array}{c} x_{i}^{n}=\{\begin{array}{cc} x_{i}^{n}\left[2\right]=\left\{34,40\right\}, & n=9\;\;\text{or}\;\;10\\ x_{i}^{n}\left[5\right]=\left\{20,30,40,60,120\right\}, & n=11\;\;\text{or}\;\;12. \end{array} \end{array}$$

By the definitions of the particle position, each particle represents a feasible solution of the impedance mismatch problem in the IPSO-IMp.

*Velocity*. The velocity of particles is defined as the change of particle position. The velocity vector of each particle *i* is represented by

(21) $$\begin{array}{c} V_{i}=\left[v_{i}^{1},v_{i}^{2},v_{i}^{3},\ldots ,v_{i}^{12}\right], \end{array}$$

where *v*_*i*_^*n*^ is defined as the change of *X*_*i*_^*n*^  (1 ≤ *n* ≤ 12).

#### 3.3.2. Velocity Updating

The range of each parameter is different, where 1 ≤ *n* ≤ 8 are successive, while the four parameters of 9 ≤ *n* ≤ 12 are discrete. Thus, the velocity updating is according to the following:

(22) vi_k+1n={vi_kn+c1r1(Pbestin−xi_kn) +c2r2(Gbest−xi_kn),1≤n≤8,−1,if  (Pbestin<xi_kn)&&(Gbest<xi_kn),9≤n≤12,1,if  (Pbestin>xi_kn)&&(Gbest>xi_kn),9≤n≤120,if  (Pbestin<xi_kn<Gbest)  ||(Gbest<xi_kn<Pbestin), 9≤n≤12.

In (22), *k* denotes iteration *k* · *v*_*i*_*k*_^*n*^ and *v*_*i*_*k*+1_^*n*^ represents the velocity of particle *i* at iteration *k* and *k* + 1, respectively. *c*_1_ and *c*_2_ are the weight of local optimal and global optimal. *r*_1_ and *r*_2_ are the random numbers. *P*_best_*i*__^*n*^ denotes the local optimal found by particle *i* until iteration *k* · *G*_best_ denotes the global optimal by the neighbors of particle *i* · *x*_*i*_*k*_^*n*^ represents the position of particle *i* at iteration *k*.

#### 3.3.3. Position Updating

According to velocity updating, the position updating in this paper adopts Algorithm 1, where *k* denotes iteration *k*.

#### 3.3.4. Avoid Local Optimal

According to dynamic behavior of the particles, the optimal solution will tend to a specific value after several iterations, which indicates that particle swarm has been or will be trapped into local optimal state according to the existing trajectory. The global optimal value, which reflects the current state of the particle swarm, could be adopted to conduct adaptive perturbation. Since the fitness function proposed aims to find the minimum value after successive *N* iterations, there is

(23) $$\begin{array}{c} G_{\text{best},\text{avg}}=\frac{1}{N}\overset{N}{\underset{i=1}{\sum }}G_{\text{best},i}, \end{array}$$

where *G*_best,*i*_ is the global optimum in iteration *i*. If *G*_best,*N*+1_ = *G*_best,avg_, the algorithm is probably stagnant, which means particles cannot escape from the local optimum. And thus particle perturbation mechanism is required for intervention.

In summary, the algorithm IPSO-IMp includes the following steps.

**Step 1**

Define individual particles of 12-dimensional space by taking the 12 parameters of fitness function as elements, and particle swarm size is set to *M*.

**Step 2**

Initialize population particles randomly according to the constraint range of 12 parameters.

**Step 3**

Calculate adaptive value for each particle according to the fitness function established by (18) and record the individual and global optimal value.

**Step 4**

Update velocity and position for each particle in accordance with (22) and Algorithm 1, aiming to seek the minimum value for the fitness function.

**Step 5**

Calculate adaptive value for each particle again according to (18) and update the global optimal value.

**Step 6**

According to the global optimal value, judge the particle aggregation state by (23). If *G*_best,*N*+1_ = *G*_best,avg_ is satisfied, particles are trapped into local optimum and then the perturbation mechanism [11] is adopted to stimulate particle energy dynamically. If *G*_best,*N*+1_ ≠ *G*_best,avg_ is satisfied, the individual optimum is update.

**Step 7**

Judge whether the iterative precision or the number of iterations are reached. If not, turn to Step 4. If so, the optimal solution is obtained and the optimization process is finished.

## 4. Experimentation and Simulation

The simulation experiment is conducted on multicore DSP processor and DDR3 interconnect bus, taking IBIS model of TI TMS320C6678 and Samsung K4B4G1646B as simulation models, respectively. Considering typical routing of PCB and the signal quality, the transmission line connecting DSP and DDR3 is abstracted into 2 vias and 3 sections of transmission line. The length of these transmission lines is *L*_1_, *L*_2_, and *L*_3_, respectively, and their impedance is *Z*_*L*_1__, *Z*_*L*_2__, and *Z*_*L*_3__, respectively.

To be consistent with the actual design, the specific parameters for simulation are listed in Table 2.

### 4.1. Optimization Result

To illustrate the performance of IPSO-IMp algorithm, the standard PSO, IPSO [11], and IPSO-IMp are, respectively, used to optimize the 12 parameters of the fitness function established in this paper.

**Figure 6:**
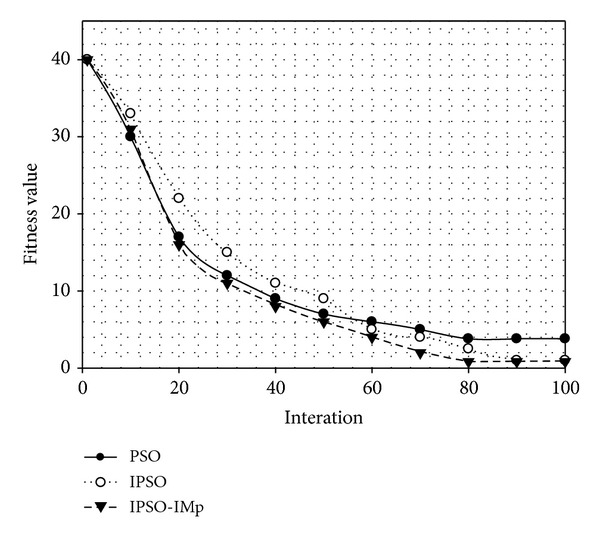
Comparison of different optimization algorithms.

The comparison is shown in Table 3. The IPSO-IMp and IPSO reveal obvious advantage in both speed and accuracy compared to standard PSO. The standard PSO and IPSO-IMp roughly perform equal in early stage of the optimization. Yet, standard PSO is in a state of local aggregation during later stage. In that case, particle movement is restricted in a small local area, so the optimal regional value cannot be searched. Because of effective perturbation, the IPSO algorithm makes the particles still maintain a certain activity in later stage. Performance comparison chart of the three optimization algorithms is shown in Figure 6.

IPSO-IMp algorithm guarantees the efficiency of early optimization. In later optimization, once the particles are found in local optimal aggregation, the movement perturbation is launched, from which particles are activated, founding the better value around and obtaining global optimum. Although the optimization results of IPSO and IPSO-IMp are approximately equal, the iteration speed of IPSO-IMp is faster nearly 12% than IPSO. That is because the latter introduces perturbation too early, resulting in increasing of the number of iterations. Compared to the standard PSO algorithm IPSO-IMp improves the accuracy significantly and the speed increases nearly 33%.

In addition, the same circuit is optimized using the approach given in [4] in order to illustrate the advantages of our method. Similarly, the routing between DSP and DDR3 is divided into 3 sections. The optimization results are shown in Table 4.

When the radius and the length of via are less than or equal to 6 mil and 1.6 mm, respectively, the influence of continuity in signal transmission changes slightly. For the actual design of 12 layers when considering the plate making technology, the via radius *r*_via_ is assigned to 6 mil and the via length *h*_via_ is assigned to 1.6 mm; namely, the thickness of board is 1.6 mm.

### 4.2. Validations and Analysis

ADS simulation software is used to verify validity of the optimized parameters from two aspects, frequency domain and time domain. Reading and writing circuits need to be designed, respectively, in our experiments, with parameters configured according to Table 4. Values of routing parameters and via parameters in both circles are identical, while the source resistance and the load resistance are assigned to their best values when reading and writing.

#### 4.2.1. Frequency Domain Analysis

**Figure 7:**
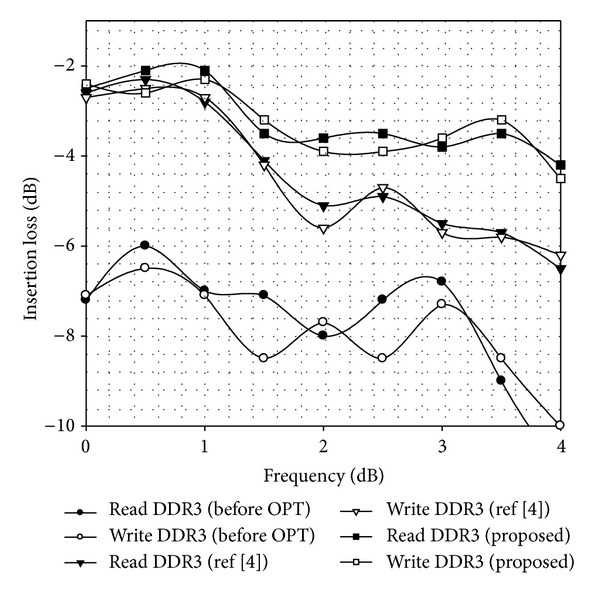
Simulation results for frequency domain.

During the transmission of high-speed signal, transmission constant should be close to 0 dB if impedance of transmission path is completely matched. Yet, due to the influence of many factors such as routing density and via parasitic effect, impedance discontinuity in transmission path emerges, leading to signal reflection and thus signal loss. Simulation results in frequency domain are shown in Figure 7.

The preoptimized design (before OPT) does not consider various factors causing impedance discontinuity, which mainly comes from improper selection of values in transmission lines, vias parameters, and ODT; thus, the signal at the receiving end suffers from serious loss. According to simulation result after optimization, we observe that our method (proposed) obviously reduces signal loss compared with the strategy presented in [4]. Our method takes the parasitic effect of via as one of optimization parameters, while [4] considers transmission line only without taking via into account. Hence, via effect is not obvious and the signal quality is roughly equal in low frequency (<1 GHz). Yet, optimization strategy we proposed displays distinct advantages with the increasing of frequency, especially when the frequency is beyond 1.5 GHz. When the frequency is 1.6 GHz, the method we proposed achieves better signal quality than [4] by 1.3 dB. Furthermore, the experimental results show that the advantage will be more obvious as the signal frequency increases.

The main reason involves two aspects. Firstly, the parasitic effect of via is not obvious in low frequency. Yet, the impact of via on signal reflection starts manifesting with the increasing of frequency. Due to proper selection of via parameters in the signals optimization, the characteristic impedance of the transmission line achieves matching, which improves the impedance continuity. Secondly, IPSO-IMp obtains better accuracy than standard PSO.

#### 4.2.2. Time Domain Analysis

**Figure 8:**
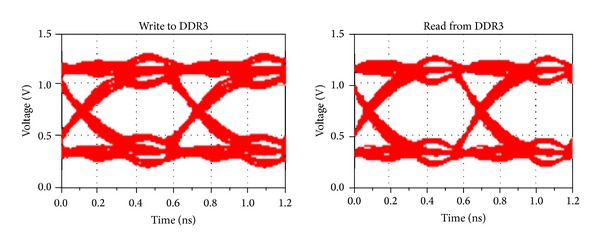
Simulation eye diagram for the general design.

**Figure 9:**
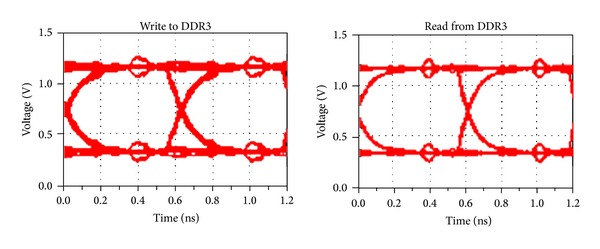
Simulation eye diagram for the method of [4].

In order to analyze the effectiveness of our optimization strategy in the time domain, this section achieves the eye diagram simulation of DDR3 reading and writing circuits. The data rate is set to 1.6G bps and each data line transmits random number. The simulation eye diagram before optimization is shown in Figure 8. It can be observed that there are overshoots and undershoots, which indicates that signal reflection exists due to impedance discontinuities and the quality of eye diagrams is poor.  Figure 9 is the simulation eye diagram when using the strategy in [4]. The quality gains effectively improvement compared with that before optimization. The signal reflection is significantly weakened, with the eye diagram higher by 155 mV averagely when reading and writing.

**Figure 10:**
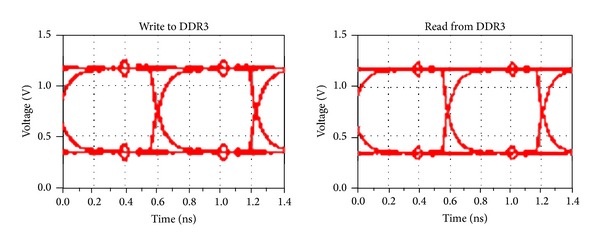
Simulation eye diagram for the proposed method.

When adopting the approach we proposed, the simulation eye diagram of DDR3 reading and writing circuits is given in Figure 10. As is shown, the eye quality is improved even more obviously. Compared to the former optimization, the height of the eye diagram improved 173 mV averagely. The detailed result of the eye diagrams simulation is listed in Table 5.

## 5. Conclusions

This paper presented an enhanced optimization strategy for DDR3 high-speed bus design, which aims to reduce the signal reflection. The strategy obtains parasitic effects of via specifically for equivalent circuit model through theoretical derivation. Additionally, we proposed an improved particle swarm algorithm to optimize the parameters in high-speed bus design. The experiments of frequency domain and time domain demonstrate that the strategy proposed could improve effectively impedance continuity of transmission line and reduce reflection effects of high-speed signals. The superiority is even more obvious with the increasing data transfer rate. It provides the referential meaning for the design of DDR4 and even higher speed bus.

## Figures and Tables

**Algorithm 1:**
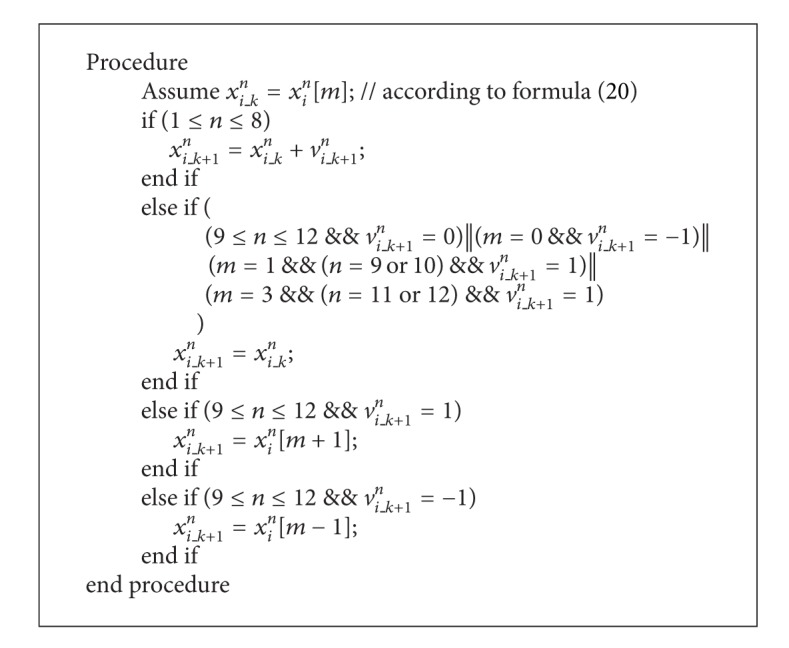
Pseudocode for the position update procedure.

**Table 1:** The parameters in the fitness function.

No.	Name	Description	Range
1	*L*_1_	Length of transmission lines	0 ≤ *L*_*n*_ ≤ 2000 mil
2	*L*_2_		
3	*L*_3_		

4	*Z*_*L*_1__	Impedance of transmission lines	20 Ω ≤*Z*_n_≤ 80 Ω
5	*Z*_*L*_2__		
6	*Z*_*L*_3__		

7	*r*_via_	Radius of the via	0 ≤ *r*_via_ ≤ 20 mil

8	*h*_via_	Length of the via	0 ≤ *h*_via_ ≤ 20 mil

9	*R*_*SW*_	Value of driver resister in writing	*R*_*SW*_ = {34,40}

10	*R*_*SR*_	Value of driver resister in reading	*R*_*SR*_ = {34,40}

11	*R*_*LW*_	ODT in writing	*R*_*LW*_ = {20,30,40,60,120}

12	*R*_*LR*_	ODT in reading	*R*_*LR*_ = {20,30,40,60,120}

**Table 2:** Design and simulation of parameters.

Parameter	Value
IPSO-IMp	
Swarm size	50
Iteration	100
Inertia weight	0.4~0.9
Constant acceleration	2
Signal parameter	
Amplitude	1.5 V
Data rate	1.6 Gbps
Rise time	50 ps
Other	
Permittivity	4.5
Stackup thickness	0.8 mm
Line space	5 mil

**Table 3:** Comparison of different optimization algorithms.

Algorithm	Best fitness (*F*_min⁡_)	Cost time (s)
PSO	3.5247	71
IPSO	0.9125	63
IPSO-IMp	**0.8932**	**48**

**Table 4:** The results of parameter optimization.

Parameter	This paper	Reference [4] method
Line length		
*L*_1_	200 mil	346 mil
*L*_2_	1356 mil	951 mil
*L*_3_	128 mil	581 mil
Impedance		
*Z*_*L*1_	48 Ω	44 Ω
*Z*_*L*2_	45 Ω	47 Ω
*Z*_*L*3_	52 Ω	55 Ω
Write DDR3		
*R*_*SW*_	34 Ω	34 Ω
*R*_*LW*_	60 Ω	60 Ω
Read DDR3		
*R*_*SR*_	40 Ω	40 Ω
*R*_*LR*_	60 Ω	60 Ω
The radius of the via (*r*_via_)	6 mil	—
The length of the via (*h*_via_)	1.6 mm	—

**Table 5:** The details of eye diagrams.

	Write to DDR3	Read from DDR3
General design		
Magnitude (mV)	424.52	428.37
Width (ps)	560.34	548.21
Reference [4]		
Magnitude (mV)	584.14	579.36
Width (ps)	577.44	572.62
**Proposed**		
** Magnitude (mV)**	**601.03**	**598.01**
** Width (ps)**	**584.51**	**581.60**

## References

[1] Jagdale R, Reddy A, Sundeep K Optimization of reflection issues in high speed printed circuit boards.

[2] Mintarno E, Ji SY Bit-pattern sensitivity analysis and optimal on-die-termination for high-speed memory bus design.

[3] Lin D-B, Houng M-P, Liu W-S Enhancement of signal integrity for multi-module memory bus by particle swarm optimization.

[4] Lin D-B, Wu F-N, Liu W-S, Wang C-K, Shih H-Y (2011). Crosstalk and discontinuities reduction on multi-module memory bus by particles warm optimization. *Progress in Electromagnetics Research*.

[5] Pan S, Fan J (2012). Characterization of via structures in multilayer printed circuit boards with an equivalent transmission-line model. *IEEE Transactions on Electromagnetic Compatibility*.

[6] Wu S, Fan J (2012). Analytical prediction of crosstalk among vias in multilayer printed circuit boards. *IEEE Transactions on Electromagnetic Compatibility*.

[7] Xuanwei C, Yu T, Ling T Electromagnetic characterization analysis of the connecting structure of the via in multilayered microwave circuit.

[8] Jiang Y, Hu T, Huang C, Wu X (2007). An improved particle swarm optimization algorithm. *Applied Mathematics and Computation*.

[9] Yau H-T, Lin C-J, Liang Q-C (2013). PSO based PI controller design for a solar charger system. *The Scientific World Journal*.

[10] Pehlivanoglu YV (2013). A new particle swarm optimization method enhanced with a periodic mutation strategy and neural networks. *IEEE Transactions on Evolutionary Computation*.

[11] Li W-T, Shi X-W, Hei Y-Q (2008). An improved particle swarm optimization algorithm for pattern synthesis of phased arrays. *Progress in Electromagnetics Research*.

[12] Melin P, Olivas F, Castillo O, Valdez F, Soria J, Valdez M (2013). Optimal design of fuzzy classification systems using PSO with dynamic parameter adaptation through fuzzy logic. *Expert Systems with Applications*.

[13] Maldonado Y, Castillo O, Melin P (2013). Particle swarm optimization of interval type-2 fuzzy systems for FPGA applications. *Applied Soft Computing*.

[14] Ying-Qiu L, Yu-Hong C, Tao W (2013). A dynamic boundary based particle swarm optimization. *Acta Electronica Sinica*.

[15] Kwon D, Kim J, Kim K (2003). Characterization and modeling of a new via structure in multilayered printed circuit boards. *IEEE Transactions on Components and Packaging Technologies*.

[16] Wu B, Tsang L (2009). Modeling multiple vias with arbitrary shape of antipads and pads in high speed interconnect circuits. *IEEE Microwave and Wireless Components Letters*.

[17] Krishna KS, Bhat MS (2012). Minimization of via-induced signal reflection in on-chip high speed interconnect lines. *Circuits, Systems, and Signal Processing*.

